# Expatiating the Pharmacological and Nanotechnological Aspects of the Alkaloidal Drug Berberine: Current and Future Trends

**DOI:** 10.3390/molecules27123705

**Published:** 2022-06-09

**Authors:** Tapan Behl, Sukhbir Singh, Neelam Sharma, Ishrat Zahoor, Ali Albarrati, Mohammed Albratty, Abdulkarim M. Meraya, Asim Najmi, Simona Bungau

**Affiliations:** 1 Chitkara College of Pharmacy, Chitkara University, Rajpura 140401, Punjab, India; singh.sukhbir12@gmail.com (S.S.); neelam.mdu@gmail.com (N.S.); ishratzahoor07@gmail.com (I.Z.); 2 Rehabilitation Health Sciences College of Applied Medical Sciences, King Saud University, Riyadh 145111, Saudi Arabia; albarrati@ksu.edu.sa; 3 Department of Pharmaceutical Chemistry and Pharmacognosy, College of Pharmacy, Jazan University, Jazan 45142, Saudi Arabia; malbratty@jazanu.edu.sa (M.A.); ameraya@jazanu.edu.sa (A.N.); 4 Pharmacy Practice Research Unit, Department of Clinical Pharmacy, College of Pharmacy, Jazan University, Jazan 45124, Saudi Arabia; anajmi@gmail.com; 5 Department of Pharmacy, Faculty of Medicine and Pharmacy, University of Oradea, 410028 Oradea, Romania; simonabungau@gmail.com; 6 Doctoral School of Biomedical Sciences, University of Oradea, 410087 Oradea, Romania

**Keywords:** berberine, isoquinoline alkaloid, antihypertensive, non-alcoholic fatty liver disease, antidiabetic, anti-inflammatory, anti-obesity

## Abstract

Traditionally, herbal compounds have been the focus of scientific interest for the last several centuries, and continuous research into their medicinal potential is underway. Berberine (BBR) is an isoquinoline alkaloid extracted from plants that possess a broad array of medicinal properties, including anti-diarrheal, anti-fibrotic, antidiabetic, anti-inflammatory, anti-obesity, antihyperlipidemic, antihypertensive, antiarrhythmic, antidepressant, and anxiolytic effects, and is frequently utilized as a traditional Chinese medicine. BBR promotes metabolisms of glucose and lipids by activating adenosine monophosphate-activated protein kinase, stimulating glycolysis and inhibiting functions of mitochondria; all of these ameliorate type 2 diabetes mellitus. BBR has also been shown to have benefits in congestive heart failure, hypercholesterolemia, atherosclerosis, non-alcoholic fatty liver disease, Alzheimer’s disease, and polycystic ovary syndrome. BBR has been investigated as an interesting pharmacophore with the potential to contribute significantly to the research and development of novel therapeutic medicines for a variety of disorders. Despite its enormous therapeutic promise, the clinical application of this alkaloid was severely limited because of its unpleasant pharmacokinetic characteristics. Poor bioavailability, limited absorption, and poor water solubility are some of the obstacles that restricted its use. Nanotechnology has been suggested as a possible solution to these problems. The present review aims at recent updates on important therapeutic activities of BBR and different types of nanocarriers used for the delivery of BBR in different diseases.

## 1. Introduction

Over the years, natural compounds, usually obtained from plants, have been commonly employed for the diagnosis and treatment of a wide range of disorders. Plants have a broad range of medicinal and biological functions, and their superior safety, availability, accessibility, tolerance, and minimum toxicity make them an ideal source for drug development, with promising therapeutic benefits [1,2,3]. Alkaloids, tannins, terpenoids, flavonoids, and steroids are secondary metabolites of plants that have a variety of pharmacological actions [4]. Alkaloids are a class of naturally occurring substances that have been utilized in traditional therapies for decades and are now being used in modern medicine as well [5,6]. Isoquinoline alkaloids are the second most common class of plant-based alkaloids, having anti-inflammatory, analgesic, antimicrobial, anti-tumor, antioxidant, anti-tussive, and narcotic functions [7]. Berberine (BBR) is a quaternary benzylisoquinoline natural alkaloid that has been used in Chinese and Ayurvedic medicine since ancient times. BBR is found in the rhizomes, roots, and bark of the stems of a variety of medicinal plants, such as *Berberis aquifolium* (Oregon grape), *Hydrastis canadensis* (goldenseal), *Coptis berberis* (coptis or golden thread), *Berberis vulgaris* (barberry), and Indian berberis, i.e., *Berberis aristata* (tree turmeric). BBR, palmatine, and berbamine are among its bioactive ingredients. *Berberis aristata* (shrub) is the most common source of BBR; it develops up to a height of 1.5–2.0 m and is indigenous to the Himalayas, where it may be found at heights ranging from 2000 to 3500 m. In southern India, it is also present in the Nilgiri Range. This shrub possesses cylindrical-shaped leaves with very sharp, tapering smooth spines and many yellow-colored stalked flowers, which are grouped in drooping racemes, as well as small berries that are smooth and oval-shaped [8,9,10]. BBR is linked to a class of chemical substances widely recognized as protoberberine alkaloids and derivatives, which have a protoberberine moiety composed of a 5,6-dihydrodibenzene molecule linked with a nucleus of quinolizinium, resulting in a 5,6-dihydrodibenzo(a,g) quinolizinium skeleton [11,12,13]. BBR has an antimicrobial effect against a wide range of species, such as fungi, chlamydia, bacteria, protozoans, helminthes, and viruses, and is useful in the therapy of a variety of skin and eye conditions. It also possesses antimalarial, cerebro-protective, anti-hyperglycemic, antifungal, antihypertensive, antioxidative, anti-inflammatory, antiarrhythmic, and anti-tumor properties. It was recently discovered that BBR is actively involved in the reduction of both fat accumulations and cholesterol in the plasma and the hepatic cells [14,15,16]. These possible biological functions of BBR and its known metabolites were believed to be due to their capacity to bind to a diverse spectrum of biological targets engaged in the etiology of different disorders [17]. 

The present review highlights the importance of nanotechnology in the delivery of BBR with improved bioavailability and increased therapeutic efficacy after considering its low water solubility and limited gastrointestinal absorption. Furthermore, this review will draw formulation scientists’ attention to the concerns and obstacles connected with its delivery as well as the potential techniques that may be explored to help patients reap the maximum benefit from this potentially useful drug. This would increase awareness among research scientists to understand better future research and development in the field of nanocarrier-based delivery of different herbal drugs.

## 2. Berberine: Chemical Properties and Pharmacokinetic Profile

**Figure 1:**
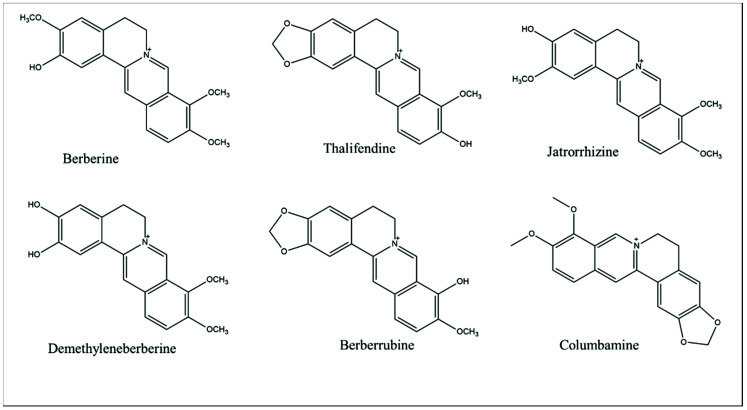
Chemical structure of berberine and its bioactive components.

Berberine is a solid, bright yellow-colored chemical that is practically water-insoluble, with a 145 °C melting point, bitter taste, and neutral nature. For therapeutic purposes, it is usually given as a chloride or sulfate salt [18,19]. The application of BBR is, unfortunately, restricted because of its low oral bioavailability. P-glycoprotein is believed to play a role in BBR’s low intestinal absorption; thus, P-glycoprotein inhibitors may be useful in improving bioavailability. BBR has been found in the urine, plasma, and bile of rabbits when given at a concentration of 2 mg/kg [20]. BBR has a complicated pharmacokinetic–pharmacodynamic (PK–PD) relationship after intravenous delivery. BBR was quickly removed from the plasma of the rat, with 1.13 h of t_1/2_ (half-life); however, BBR (3 mg/kg, i.v.) in the hippocampus of the rat rose sharply (t_1/2_ = 0.215 h), reached its peak at 3.67 h, and had a delayed clearance rate (t_1/2_ = 12.0 h), indicating that BBR may act instantly on neurons and be stored in the hippocampus [21]. BBR (3 mg/kg, i.v.) was also found in abundance in the thalamus area of the brain of rats and has a 14.6 h clearance half-life. This suggests that BBR has a different pharmacokinetic profile in the thalamus or hippocampus and plasma in rats [22]. Furthermore, following oral delivery, the levels of BBR and its bioactive metabolic products in organs were shown to be greater than those in the blood. BBR has a fast organ distribution, with the highest concentration in the liver, followed by kidneys, muscle, lungs, brain, heart, and pancreas, and the lowest concentration in fat, which remains generally stable for 48 h [23]. BBR is metabolized in the liver of both humans and rats, where it undergoes phase I demethylation before combining with sulfuric acid or glucuronic acid to produce phase II metabolites. The polar sulfate or glucuronide metabolites produced are easily eliminated [24]. Phase II metabolites included “glucuronic acid-conjugated: thalifendine, berberrubine, jatrorrhizine, and demethylene berberine metabolites; sulfate-conjugated: berberrubine and thalifendine; and diglucuronide-conjugated demethylene berberine”. BBR and its metabolic products were found in considerable quantities in the feces (84%). In the bile, BBR was predominantly eliminated as thalifendine, with thalifendine and berberrubine accounting for 78% of urine excretion [25]. As a result, rodents could be used to test the therapeutic effects of BBR. The various pharmacological actions of BBR indicated that it could be used as a therapeutic for a number of clinical purposes. In this regard, BBR shows a biologically significant skeleton as well as an appealing natural lead molecule for the insertion of numerous chemical changes in proper sites in the quest for even better selective, distinguished, and restricted therapeutic indication. Figure 1 depicts the chemical structure of BBR and bioactive components. Table 1 summarizes the physicochemical properties as well as the natural sources of BBR [26,27,28]. 

## 3. Therapeutic Roles of Berberine

**Figure 2:**
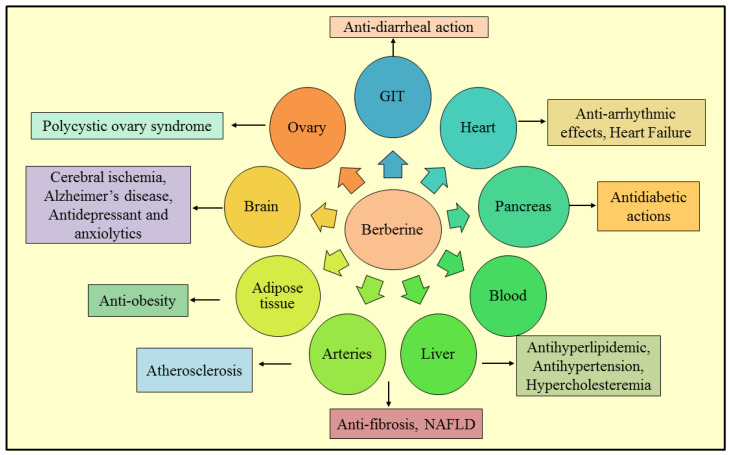
Schematic representation of various pharmacological actions exerted by berberine on different organs in the human body. NAFLD—non-alcoholic fatty liver disease, GIT—gastrointestinal tract.

Berberine can easily pass the blood–brain barrier when administered systemically. Preclinical research has indicated that it may be effective in treating a number of neuropsychiatric and neurodegenerative conditions. It also possesses a broad spectrum of therapeutic properties, such as antihypertensive, antibacterial, anti-tumor, anti-HIV, and anti-inflammatory properties. Anti-cholinergic, anti-protozoal, cardiotonic, cholagogic, antiarrhythmic, choleretic, and anti-platelet aggregation properties are also present. The following sections highlight several therapeutic properties of BBR that have been widely researched [29]. Figure 2 depicts the potential pharmacological activities of BBR.

### 3.1. Non-Alcoholic Fatty Liver Disease

Non-alcoholic fatty liver disease (NAFLD) is a frequent disorder during which large amounts of fat accumulate in the liver of individuals who consume little or do not drink alcohol. NAFLD is strongly associated with obesity and other metabolic conditions (dyslipidemia and T2DM). NAFLD can develop into non-alcoholic steatohepatitis, which is marked by chronic inflammation of hepatocytes and damage, with or without fibrosis, if left untreated [30,31]. The initial therapy approach for NAFLD is to restore dysregulated metabolism of lipids, which results in fat loss and weight loss [32]. In both clinical trials and animal models, BBR has been shown to be a promising treatment for NAFLD. BBR significantly lowers fat accumulation in the hepatic cells in hyperlipidemic hamsters, and it also decreased liver steatosis and reduced 14% of liver lipid content in mice on a high-fat diet [33]. It has also been clearly established that BBR lowers liver necrosis in both non-alcoholic and hepatitis C infection-related steatosis [34]. 

### 3.2. Anti-Diarrheal Action

Berberine has been utilized in the therapy of diarrhea for decades in various regions of the globe, and it is still used today. Its anticholinergic, antimicrobial, and/or anti-secretory properties, as well as its α2-adrenoreceptor agonistic properties, provide evidence of its activity in this regard. It has also been reported to inhibit several enterotoxins produced by *V. cholera* and *E. coli* in humans, to decrease the contraction of smooth muscle and motility, and to delay the transit time of intestines. BBR sulfate works by preventing bacteria from adhering to epithelial or mucosal surfaces, which is the first step in the infection process. This could be due to its inhibitory effect on the formation of fimbrial structures on the membrane of treated bacteria. BBR significantly prevented both castor oil- and BaCl_2_-induced diarrhea in animals when given orally at doses greater than 25 mg/kg but not pilocarpine- or serotonin-induced diarrhea, even at 250 mg/kg. BBR prevented Ba^2+^ or acetylcholine-induced ileum and colon contractions when used at doses of 5–10 g/mL [35,36].

### 3.3. Anti-Fibrotic Effect

The uncontrolled growth of connective tissue in the liver is known as hepatic fibrosis, and it is caused by a variety of pathogenic conditions. The therapy of hepatic fibrosis involves a number of strategies, including suppressing the growth of hepatic stellate cells (HSC), increasing collagen breakdown, and many others [37]. BBR has been found to have a number of anti-fibrotic properties. BBR inhibits the activation of HSC, which is a crucial step in the progression of hepatic fibrosis, by inducing HSC cycle arrest in the G1 phase. BBR decreased the phosphorylation of FoxO1(forkhead) box O1 and Akt in rat liver fibrosis produced by bile duct ligation, as well as in HSC [38,39]. BBR also stimulated the AMP-activated protein kinase (AMPK) signaling pathway, which reduced the levels of Nox4 and p-Akt, both of which have been linked to liver fibrosis [40]. BBR also prevented the expression of transforming growth factor (TGF-β1), and α-smooth muscle actin (α-SMA), which are markers of activated HSC transitioning from quiescence to myofibroblast (MFB)-like cells, and thereby reversed liver fibrosis [41]. BBR reduced hepatic fibrosis by stimulating collagen breakdown through lowering tumor necrosis factor-alpha (TNF-α) and TGF-β1 expressions and enhancing matrix metalloproteinase (MMP)-2 concentrations. The pharmacological effectiveness of BBR against hepatic fibrosis was contributed to by these various mechanisms [42,43].

### 3.4. Alzheimer’s Disease

Alzheimer’s disease (AD) is a serious neurological illness that impacts a growing number of aging people and progressively deteriorates memory and other essential brain functions. Its symptoms appear gradually, impact the brain, and are degenerative, which means that they induce gradual degeneration [44,45]. Numerous variables have been found to be involved in the etiology of AD, providing varied targets for therapeutic screening, such as acetylcholinesterase enzyme (AChE), monoamine oxidase (MAO), butyrylcholinesterase, oxidative stress, amyloid-peptide (A) aggregation, and others. BBR is believed to be a therapeutic option for preventing and delaying the onset of AD by restricting the function of these risk variables and reducing the metabolic syndrome related to the condition [46,47].

### 3.5. Antidepressant and Anxiolytic Effects

Depression is a medical condition marked by a depressed mood—a lack of interest or pleasure in normal actions—resulting in considerable impairment in normal life, and has been identified as one of the world’s most serious medical disorders. Antidepressants are the drugs used in the treatment of depression, dysthymia, social anxiety disorder, and other mental disorders. Antidepressants that block MAO are the most common type. MAO is an enzyme that causes the breakdown of neurotransmitters in neural tissue, including dopamine, noradrenaline, and serotonins, which become insufficient in the brain and cause depression. MAO inhibitors enhance the levels of important neurotransmitters within the brain, resulting in a significant enhancement in the pharmacological therapy of depression. BBR inhibits MAO activity, which helps to relieve depressive symptoms [48,49,50]. BBR (100 mg/kg) shows a positive anxiolytic action through stimulating 5-HT1A receptors and blocking postsynaptic 5-HT2 and 5-HT1A receptors, which could be linked to greater monoamine production levels within the brain stem and lower serotonergic system function [51].

### 3.6. Cerebral Ischemia

Since microcirculation dysfunction is a major pathophysiological alteration in ischemic cerebrovascular disorder, improving cerebral pial microcirculation is essential for maintaining cerebral blood circulation and treating cerebral ischemia symptoms. BBR has a vasodilator effect, significantly dilating the cerebral pial microcirculation peripheral arteries, increasing microcirculation blood flow, and regulating blood circulation to the brain and nerve cell basic metabolism. After cerebral ischemia, BBR (20 mg/kg per day, i.p. 5 days) was found to block the increased platelet aggregation and adhesion rate caused by adenosine diphosphate, arachidonic acid, or collagen. Furthermore, BBR has been shown to reduce lesions of cerebral vascular endothelial cells, which have been associated with the incidence and progression of cerebral vascular disorders, especially in the initial stages of cerebral ischemia [52]. 

In recent research, barberry extract was found to have neuroprotective properties in the cerebral ischemic rat model. The protective effect could be explained by BBR’s inhibitory action on the receptors of N-methyl-d-aspartate (NR), specifically the NR1 subtype. High expression of NR1 receptors occurs after 30 min of focal ischemia, which can be reversed with BBR (7.45+/0.85 mg/g) pre-treatment. The anti-inflammatory characteristics of BBR were thought to be responsible for its neuroprotective effects in cerebral ischemia [53,54].

### 3.7. Anti-Obesity

Obesity is a medical disorder that causes an increase in body fat, putting individuals at risk of health problems such as heart disease, T2DM, and cancer [55,56]. BBR is a drug that could be used to treat obesity. It works by inhibiting the processes of adipogenesis and lipogenesis. BBR’s anti-obesity action is linked to the fact that it significantly decreases the number and dimensions of lipid droplets in the adipocyte cell line. BBR enhances the basal lipolysis condition of triglycerides in adipocytes by increasing AMPK-mediated adipose triglyceride lipase (ATGL) production, which improves the long-term body weight loss effect [57]. BBR also inhibits the differentiation and proliferation of preadipocytes. BBR reduces adipocyte differentiation by inhibiting the transcription factors peroxisome proliferator-activated receptor gamma (PPARγ) and C/EBPα, both of which are required for adipogenesis [58,59]. Experiments show that BBR can reduce the levels of cholesterol, which are linked to intestinal absorption inhibition, as well as the micellarization of cholesterol and uptake of cholesterol by enterocytes. BBR also inhibits the expression of acyl-coenzyme-A cholesterol acyltransferase-2 (ACAT2) and reduces permeability in Caco-2 monolayers, which diminishes cholesterol esterification and secretion. BBR has a lipid-lowering action by regulating the metabolism of cholesterol and bile acid homeostasis [60,61]. BBR has been shown to alter the gut microbiota composition. BBR induces gut microbiota modification in a manner similar to probiotics [62,63,64]. BBR stimulates the signaling system AMPK. AMPK performs a crucial part in the maintenance of a cell’s energy and metabolic landscape. It functions as a cellular energy state sensor. It serves as a fuel gauge and monitors cellular levels such as the AMP/ATP ratio. AMPK is a promising therapeutic target, since it controls energy homeostasis throughout the body [65].

### 3.8. Polycystic Ovary Syndrome

Polycystic ovary syndrome (PCOS) is a clinical disease characterized by reproductive and endocrine abnormalities and affects 5–10% of females of childbearing age. PCOS is linked to luteinizing hormone hypersecretion, hyperinsulinemia, hyperandrogenemia, hirsutism, menstrual dysfunction, infertility, and pregnancy and neonatal problems [66,67]. The ovaries of a female with PCOS are largely affected by the elevated levels of insulin, as it leads to increased production of androgen hormones such as testosterone. BBR increases insulin signaling pathways by increasing glucose intake through the activation of the AMPK system [68,69]. BBR has recently gained attention as a potential therapy for PCOS. According to a meta-analysis of 12 randomized controlled studies, BBR can reduce androgen levels, improve insulin resistance (IR) and dyslipidemia, and reduce the ratio of luteinizing hormone to follicle-stimulating hormone in women with PCOS [70]. BBR is effective in females with PCOS, because it lowers the free androgen index, testosterone, and androgen levels [71,72]. 

### 3.9. Antidiabetic Action

Diabetes is a long-term metabolic condition marked by elevated blood glucose levels that are caused by a lack of insulin sensitivity and pancreatic ß-cell dysfunction [73]. The most predominant type of diabetes is T2DM. Exercise and diet are the first line of treatment for T2DM, followed by oral hypoglycemic drugs and, possibly, subcutaneous insulin injections [74]. BBR is also becoming more popular due to its possible benefits for diabetic people [75]. This effect was shown to be induced by enhanced glucose homeostasis, enhanced insulin expression, regeneration of pancreatic ß-cells, and reduction in peroxidation of lipids in experimentally induced diabetic rats [76,77]. After giving BBR to individuals with metabolic syndrome, researchers found that their insulin sensitivity increased, resulting in better clinical outcomes [78,79]. The antidiabetic activity of BBR is most commonly observed in the uptake of glucose by adipose tissue and skeletal muscles as well as in hepatic gluconeogenesis. AMPK activation has been found to play a role in these activities. In mammalian cells, AMPK is considered an essential energy-sensing protein. It monitors levels of cellular energy, such as the AMP/ATP ratio, and serves as a fuel gauge. As a result, AMPK is a promising therapeutic target, since it regulates the whole-body energy balance [80]. BBR can reduce fasting blood sugar levels by activating the AMPK system through mitochondrial inhibition [81]. Moreover, BBR has the potential to restore insulin sensitivity in hepatic and muscle cells by upregulating the levels of insulin receptor (InsR) gene expression in a protein kinase D (PKD)-dependent way [82]. BBR has also been found to inhibit hepatic gluconeogenesis while promoting adipocyte differentiation, leading to a net rise in glucose utilization [83]. Recent research indicates that the gut flora may have a role in the process through which BBR lowers blood glucose and lipid levels [84,85]. BBR has been shown to be safe and effective in the treatment of patients with type 2 diabetes in clinical studies. BBR alone reduced fasting blood sugar levels by 21–36% in most cases [86]. 

### 3.10. Atherosclerosis

Atherosclerosis is a disorder marked by low-grade, persistent inflammation of the artery walls [87]. In developed nations, atherosclerosis is the main reason for mortality, with coronary heart disease responsible for the majority of heart failures. Plaque accumulation is also frequent in the peripheral system, for example, in the renal, femoral, and carotid arteries, all of which can lead to major consequences. The plaque deposition inhibits oxygenated blood from reaching downstream tissues, resulting in limb amputation, kidney failure, or stroke. Furthermore, plaque can break, and fragments can move downstream, causing the same difficulties [88,89]. BBR slowed the progression of atherosclerosis by controlling several pro-atherogenic cellular and molecular pathways. The preventive actions of BBR towards atherosclerosis may be due to the migration of vascular smooth muscle cells (VSMCs), endothelial functions, activation of platelets, and macrophage-derived foam generation [90]. VSMCs are highly specialized cells that are involved in a number of disorders, such as atherosclerosis. VSMCs, as essential elements of blood vessels, regulate the tone and diameter of vessels, both of which are important factors in regulating vascular tension and function. Different signaling pathways play a significant role in the migration, proliferation, and apoptosis of smooth muscle cells [91]. The primary reasons for the progression of atherosclerosis are increased proliferation and decreased apoptosis of VSMC. As a result, clinical treatment options include prevention of proliferation and induction of apoptosis of VSCMs [92]. In recent research, BBR was found to reduce both the multiplication and death of VSMCs caused by mechanical stretch stress, suggesting that BBR could be an effective treatment for atherosclerosis. BBR has a dual function, which provides further support for its clinical applicability. Although BBR has been shown to be useful in the treatment of atherosclerosis, its therapeutic application has been slow, and some aspects are still unknown. As the application of BBR in the area of anti-atherosclerosis develops, researchers must continue to explore it [93]. 

### 3.11. Antihyperlipidemic Effects

BBR’s metabolic actions have been extensively researched in recent years. The lipid-lowering action of BBR is believed to be mostly due to an ERK-dependent pathway that stabilizes the liver’s low-density lipoprotein-cholesterol receptor (LDLR) and to enhanced transcriptional behavior of the LDLR promoter via the c-Jun N-terminal kinase mechanism [94,95]. Furthermore, lipid production is inhibited due to the effect on 5’ adenosine monophosphate (AMP) kinase (AMPK) and the blockage of the mitogen-activated protein kinase/extracellular signal-regulated kinase (MAPK/ERK) mechanism [96]. 

The antihyperlipidemic actions of BBR have also been demonstrated in individuals through several clinical trials. Kong et al. explored the influence of BBR 500 mg in a hyperlipidemic group containing 32 Asian individuals who did not take any other medications except BBR 500 mg two times a day and compared the findings to 11 individuals who took a placebo. BBR lowered total cholesterol, triglycerides, and LDL-C by 29%, 35%, and 25%, respectively [97]. These findings were then validated in a larger study with 116 hyperlipidemic type 2 diabetic individuals who were randomly assigned to receive BBR 500 mg three times per day or a placebo. In addition to a similar reduction in plasma lipids, the BBR-treated individuals had lower glycohemoglobin, fasting blood glucose, and 2 h postprandial blood glucose levels than the placebo group [98]. 

BBR’s antihyperlipidemic action has been found to act synergistically with other nutraceuticals that inhibit cholesterol formation, such as monakolins and policosanols. Small randomized clinical research was conducted comparing 40 Caucasian, hyperlipidemic individuals randomized to receive either 500 mg/day of BBR or 500 mg of BBR given with red yeast extract (3 mg) and policosanol (10 mg/day) for four weeks. Triglyceride levels were reduced by 26% and 22% in the combination and BBR groups, respectively, while LDL-C levels were decreased by 20% and 25% in the BBR and combination groups, respectively [99]. When BBR is used with simvastatin in the treatment of hyperlipidemic individuals, a similar effect is shown [100]. 

### 3.12. Antihypertensive Activity

BBR has been proven to have vasorelaxant actions in various rat models [101,102,103]. BBR has a vasodilator effect, because it influences both the endothelium and the smooth muscle of the circulatory system. BBR-mediated aortic relaxation generally depends on endothelium at small doses (<1 × 10^−6^ M), but, at larger doses, it is independent of an intact endothelium [104,105,106]. BBR has a dose-dependent relaxing and anticonstrictive action on isolated rat thoracic aortas. BBR has shown a hypotensive action by decreasing angiotensin-converting enzyme activity and directly stimulating NO and cGMP synthesis in vascular tissues [107]. BBR decreases oxidative stress by decreasing intracellular levels of ROS, protecting endothelial cells, and improves endothelium-mediated vasodilation by lowering the expression of oxidized low-density lipoprotein (oxLDL) and TNF-α-induced lectin-like oxLDL receptor 1 [108]. 

The vasodilatory actions of BBR on methylene blue-pre-treated rat aorta were not found at small doses (below 1 × 10^−6^ M), but aortic relaxation was found at larger doses regardless of the presence of an inhibitor of nitric oxide. The above-mentioned nephroprotective function revealed in rat models could also reduce blood pressure (BP) rises [109]. BBR also has a vascular-protective effect, which may help to retain endothelial activity and keep blood vessels highly reactive and elastic. BBR prevents platelet-derived growth factor (PDGF)-stimulated VSMC development by activating the AMPK/p53/p21 (Cip1) signaling pathways, stimulating Ras/Rac1/Cyclin D/Cdks, and prevents PDGF-stimulated migration by inhibiting Rac1 and Cdc42, resulting in anti-migratory and anti-proliferative actions [110]. BBR prevents the formation of collagen and fibronectin through the p38 MAPK pathway and inhibits smooth muscle cells from migrating and regrowing by inactivating the mitogen-activated protein kinase/extracellular signal-regulated kinase/early growth response gene 1 (MEK1,2/ERK/Egr1 signaling pathway) and lowering Erg1, cFos, cyclin-D, and platelet-related growth factor A (PDGF-A) concentrations [111,112]. Proliferation and migration of VSMCs activated by lysophosphatidylcholine are suppressed by the stimulation of the ERK1/2 mechanism (one of three MAPK groups) [113]. 

In a clinical trial, BBR was reported to significantly lower patients’ mean 24 h systolic and 24 h pulse pressures [114]. BBR reduces mean and pulse BP by blocking transient receptor potential vanilloid 4 channels, lowering intracellular Ca^2+^ concentrations in VSMCs and inducing vasorelaxation [115]. Furthermore, by decreasing protein phosphatase 2A signaling mechanisms, BBR can reduce the influence of norepinephrine on pulmonary arterial hypertension [116].

### 3.13. Antiarrhythmic Effects

BBR has been proven to inhibit arrhythmias in rats and dogs. The Purkinje fibers’ effective refractory period may have increased as a result of this action. BBR has been shown to have class III antiarrhythmic properties on the heart muscle of mammals in vitro [117,118]. Most antiarrhythmic drugs of class III act by inhibiting delayed rectifier K^+^ channels of cardiac muscles. As a result, significant prolonging of myocardial refractoriness causes the activation wavelength to surpass the re-entrant circuit’s path length, prohibiting re-entrant excitation initiation and maintenance [119]. BBR may be an efficacious agent for treating atrial fibrillation, due to its various cellular pharmacological actions. Zhou et al. found that BBR reduces acetylcholine-induced atrial fibrillation in rabbits by enhancing the atrial effective refractory period and prolonging the action potential length of atrial myocytes [120]. BBR decreased the prevalence of premature ventricular beats and inhibited the onset of ventricular tachycardia in rats with stretch-induced arrhythmias following myocardial infarction [121,122]. BBR has also been demonstrated to have antiarrhythmic properties in diabetic rats. The antiarrhythmic properties of BBR may be due to its interactions with IK1/Kir2.1 [123,124]. BBR also blocks the current of hyperpolarization-activated cyclic nucleotide-gated four channels, thereby promoting pacemaker currents (Ifs) and antiarrhythmic effects [125]. 

### 3.14. Congestive Heart Failure

Congestive heart failure (CHF) develops when the muscles of the heart fail to pump blood effectively [126]. In individuals with refractory CHF, BBR improves their hemodynamic condition. BBR possesses potent inotropic and peripheral resistance-reducing properties. It decreases ventricular pressure by reducing the left ventricular systolic size and increasing the left ventricular ejection fraction. By lowering systemic and pulmonary venous pressures, BBR lowers ventricular filling pressures [127]. Calcium is essential for maintaining normal BP. In one study, BBR was found to enhance heart function by increasing calcium levels in cardiac muscle cells [128]. Because of its action on K^+^ channels, BBR raises the levels of high-energy phosphate in cardiac failure. BBR decreased the delay in depolarization caused by Na^+^ influx and prevented ventricular fibrillation. BBR also improved the contractility of cardiac muscles in experimentally induced rats by lowering plasma concentrations of noradrenaline and adrenaline as well as adrenaline levels in ventricular tissue. BBR enhances the production of nitric oxide, which improves blood flow, relaxes the arteries, decreases blood pressure, and prevents atherosclerosis [129]. BBR also lowers plasma brain natriuretic peptide levels in rats with prolonged CHF [130]. BBR has a cardiotonic and preventive action on cardiac dysfunction caused by hyperglycemia/hypercholesterolemia by reducing the accumulation of lipids in cardiac muscle cells and increasing the transport of glucose [131].

### 3.15. Hypercholesterolemia

Hypercholesterolemia is a disorder in which the blood cholesterol concentrations are abnormally high. BBR has been found to reduce levels of lipids in a variety of ways, e.g., by upregulating the expression of the LDL receptor via AMPK-dependent Raf-1 activation, downregulating PCSK9 via sterol regulatory element-binding protein-2 activation, and decreasing PDE inhibition [100,132,133,134,135,136]. BBR may also lower plasma lipids by activating AMPK, which inhibits HMG-CoA reductase activity and, therefore, decreases liver cholesterol and TG synthesis [96]. BBR’s mechanism varies significantly from that of statins, and in hyperlipidemic rats, an amalgamation of simvastatin (6 mg/kg/day, oral) and BBR (90 mg/kg/day, oral) reduced blood LDL cholesterol and serum triglycerides more effectively than BBR or simvastatin monotherapy. Therefore, combining BBR and simvastatin could be a novel hypercholesterolemia regimen [100]. In Kunming mice with streptozotocin-induced diabetes, oral dosing of BBR at 100 mg/kg/day for four weeks lowered HDL cholesterol, blood total cholesterol, and LDL cholesterol by 9%, 16%, and 20%, respectively. However, male diabetic KKAy mice given a high-calorie diet showed a significantly positive impact. BBR given orally at 250 mg/kg/day for four weeks lowered blood total cholesterol by approximately 42% [137]. Wang et al. found that BBR reduced blood cholesterol concentrations in diet-induced hypercholesterolemic rats by interacting with intraluminal cholesterol micellarization and lowering enterocyte cholesterol absorption and release as well as slowing intestinal absorption [61].

### 3.16. Anti-Inflammatory Activity

BBR has been demonstrated to have anti-inflammatory functions in a number of animal and human tissues, such as the hepatic, vascular endothelial, intestinal, and adipose tissues [138,139,140,141]. BBR inhibits the expression of several proinflammatory cytokines and acute-phase proteins, including inducible nitric oxide synthase, monocyte chemoattractant protein-1, TNF-*α*, cyclooxygenase-2 (COX-2), prostaglandins (PGs), IL-1*β*, matrix metalloprotease 9, IL-6, and C-reaction protein, all of which are essential in the prevention and treatment of inflammation-related disorders [142]. BBR has been shown to entirely inhibit TNF-α-mediated barrier deficiencies in cell models, which are linked to the tyrosine kinase, NF-κB, and pAkt pathways [143]. 

BBR was found to greatly lower the IL-6 levels in the serum of patients when given at a dose of 1 g per day for three months. BBR inhibits inflammation via a number of mechanisms. The AMPK mechanism is linked with BBR’s anti-inflammatory effect, in addition to its antioxidant properties. In macrophages, inhibiting AMPK could decrease BBR’s inhibitory action on pro-inflammatory cytokines such as Cox-2 and inducible nitric oxide synthase (iNOS) [144]. Furthermore, BBR’s anti-inflammatory properties are linked to its MAPK pathway-inhibitory action, which is dependent on macrophage AMPK activation. Further research on the significance of these interactions in the therapeutic actions of BBR is required. The NF-κB pathway is critical for inflammation control and is also a target for BBR’s anti-inflammatory effects [145]. 

## 4. Nanotechnology-Based Approaches for Berberine Delivery

**Figure 3:**
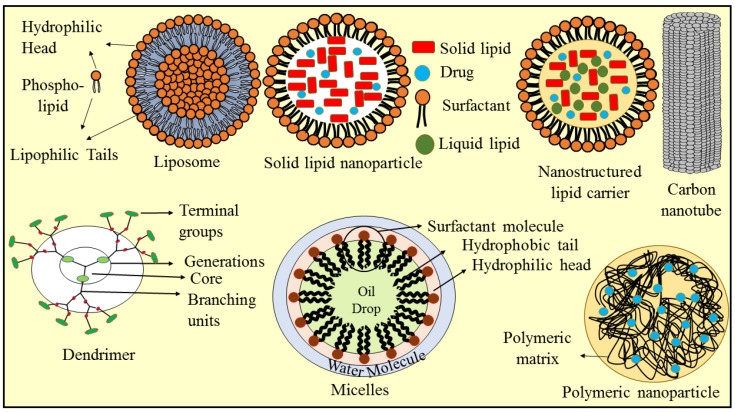
The structural representation of various types of nanocarriers explored for encapsulation of berberine molecules to overcome its therapeutic challenges.

Despite its benefits, BBR has several drawbacks in clinical use, the most significant of which is its low water solubility and limited gastrointestinal absorption. It is categorized as a class IV drug because of its poor bioavailability (about 5%). The strong binding of plasma proteins to BBR can be used to explain the poor bioavailability. Based on this, the unbound percentage of BBR that may reach and permeate target tissues is small. Furthermore, the first pass effect significantly influences BBR; this is the other factor in its poor bioavailability [146]. Considering the facts and circumstances, nanoparticulate delivery strategies are essential for mitigating these shortcomings and increasing BBR efficacy. Nanoparticles are solid particles or particulate dispersions with a diameter of 10 to 1000 nanometers. The primary objectives of nanoparticle development as a delivery method are the regulation of the size of the particles and their surface characteristics and the release of biologically active chemicals to induce site-specific drug activity at a therapeutically appropriate rate and dosing regimen [147]. Nanocarriers are particles that are designed to entrap herbal drugs in order to sustain their circulation in the bloodstream and to overcome all barriers in order that the medicine may reach its target location. Nanocarriers include micelles, carbon-based compounds, liposomes, polymers, and other materials. The use of various forms of nanomaterial in nanocarriers enables the delivery of both hydrophobic and hydrophilic molecules throughout the body [148]. Figure 3 depicts various types of nanocarriers that have been developed to entrap BBR in order to maintain its circulation in the blood and overcome all limitations, such as poor aqueous solubility, limited gastrointestinal absorption, and poor bioavailability, in order to deliver the drug to its target site. Some of the nanoparticles that have been used as BBR delivery systems are concisely mentioned in Table 2.

### 4.1. Solid Lipid Nanoparticles

Solid lipid nanoparticles (SLNs) are receiving a great deal of attention, since they offer much promise as oral drug delivery carriers [149]. As explained previously, one of the greatest challenges of BBR is its poor gastrointestinal absorption, which restricts its effectiveness. Xue et al. overcame this limitation by encapsulating BBR in SLNs, and they studied their effect in db/db mice upon oral administration. They found that SLNs were predominantly deposited in the brain, liver, and jejunum, and, from the results, it was concluded that the extensive removal of BBR was responsible for its low plasma levels. In the liver, the concentrations of triglycerides and alanine transaminase were lowered by BBR-loaded SLNs. BBR-loaded SLNs were found in the brain, indicating that these nanoparticles can cross the blood–brain barrier. The greater plasma levels and bioavailability of the BBR-loaded SLNs significantly increased their antidiabetic effects. From the results, it was revealed that BBR-SLNs (especially in the 100 mg/kg dose group) play a key role in preventing hepatic steatosis in db/db mice by inducing carnitine palmitoyltransferase-1 expression and suppressing sterol regulatory element-binding protein 1c, stearoyl-CoA desaturase 1, and fatty acid synthase expression and that the increased pharmacodynamic effects of BBR-SLNs in reducing hepatosteatosis in db/db animal models are due to the predominant concentration of BBR-SLNs in the liver [150]. Xue et al. developed BBR-SLNs in order to achieve increased bioavailability and a prolonged effect, and the SLNs exhibited homogeneous spherical shapes, a zeta potential of 7.87 mV, small size (76.8 nm), encapsulation efficiency of 58%, and drug loading of 4.2%. When compared to BBR alone (*p* < 0.05), BBR-SLNs (50 mg/kg single dose) upon oral administration demonstrated a considerable increase (*p* < 0.05) in peak plasma concentration, area under the curve, and variance of mean residence time in rats, indicating better bioavailability. Upon oral treatment, both BBR and BBR-SLNs substantially reduced body weight gain, fasting blood glucose levels, and insulin resistance homeostasis assessment as well as improving impaired glucose tolerance and insulin tolerance in db/db diabetic mice. When compared to an equivalent dose of BBR, BBR-SLNs at a high dose (100 mg/kg) were more effective. BBR-SLNs may improve islet function while also protecting it against regeneration [151]. Wang et al. evaluated the anti-tumor effect of SLNs containing BBR-hydrochloride by using nano-sized preparations against HepG2, MCF-7, and A549 malignant cells. Cell proliferation was considerably reduced in these cell lines by these nanoparticles. In this regard, cell cycle, clone development, cellular uptake, and apoptosis were all explored. The BBR-hydrochloride-loaded SLNs, as per the findings of this research, are effective drug delivery methods in cancer therapy [152].

### 4.2. Nanostructured Lipid Carriers

To solve the limitations of SLNs, a new generation of solid lipid nanoparticles, known as nanostructured lipid carriers (NLCs), was developed. In the development of NLCs, a small amount of solid lipid is substituted with liquid lipid. This creates a highly disordered lipid structure that allows for increased drug loading while also avoiding drug leakage during storage. NLCs developed from SLNs, with significant enhancements in encapsulation efficiency. The encapsulation efficiency of NLCs (93.33%) is greater than that of SLNs (75.81%) [153].

BBR has an anti-hepatocarcinoma action, which is one of its most important characteristics. Hepatocellular carcinoma is one of the most common malignancies worldwide, with a high mortality rate; therefore, increasing BBR potency in order to treat it would be quite advantageous [154]. In this respect, Meng et al. designed BBR-loaded NLCs and found that BBR-NLC efficiently reduced the development of HepG2, Huh7, and EC9706 malignant cells of humans in vitro and also demonstrated that the potential of NLCs to improve the effectiveness of BBR was likely mainly due to increased bioavailability and blood levels [155]. Wang et al. prepared BBR-loaded NLCs and observed that BBR-NLCs considerably reduced H22 cell proliferation in vitro and showed significant anticancer action in in vivo models with respect to bulk BBR solution [156]. 

Raju et al. developed BBR-NLCs by melt-emulsification and ultrasonication that were optimized by 32 factorial design, and the optimized BBR-NLCs exhibited a particle size of 186 nm, a polydispersity index of 0.108, a zeta potential of −36.86 mV, and 88% entrapment efficiency. Batch-B5 released 82% of its BBR in 24 h in phosphate buffer, and the findings of comparative pharmacodynamic studies in Albino Wistar rats revealed that BBR-NLCs enhanced behavioral parameters in vivo as compared to pure BBR [157]. Gendy et al. formulated NLC-BBRs which exhibited superior efficiency to unformulated medication, and the anti-inflammatory effect of NLC-BBRs before hepatic I/RN may be attributed to their ability to increase autophagosome flux biomarkers and suppress HMGB1, TLR4, NF-κB, TNF-α, iNOS, COX-2, and MPO. Reducing MDA and Bax and boosting TAC and Bcl-2 support its antioxidative/antiapoptotic properties, and all of these diverse preventive measures combine together to eliminate the possibility of hepatic I/RN upshots [158].

### 4.3. Liposomes

Liposomes are tiny artificial vesicles with a spherical form made of cholesterol and non-toxic phospholipids. Liposomes are potential drug carriers due to their size and hydrophilic and hydrophobic properties. Liposomes have been used as effective nanocarriers for anticancer, antifungal, antibiotic, and anti-inflammatory medicines in a variety of clinical trials [159]. Calvo et al. developed BBR-loaded liposomes and demonstrated that BBR liposomes increased the accumulation of BBR in the hepatic cells and spleen. Liposomal administration decreased parasite load in hepatic cells and spleen by 3 and 1 logarithms (99.2 and 93.5%, respectively), but free BBR only reduced infection in hepatic cells by 1 logarithm. The antileishmanial action of free BBR was increased via BBR liposome administration [160]. Lin et al. encapsulated BBR in liposomal nanovesicles and studied the activity of these nanocarriers in human liver cancer, and the in vitro investigations revealed that liposomal BBR preparations suppressed the development of HepG2 cells by inducing apoptosis via the caspase/mitochondrial-dependent mechanism and significantly decreased tumor weight and size as well as significantly inhibiting BBR clearance in plasma and tissues [161]. Allijn et al. created BBR-loaded liposomes using ethanol injection and characterized them, and, from the results of in vitro study, it was found that free BBR significantly inhibited IL-6 secretion (IC50 = 10.4 μM) and preserved the cardiac ejection fraction at day 28 after MI by 64% as compared to control liposomes and free BBR. Liposomal encapsulation increased the solubility of BBR in buffer and preserved the ejection fraction after MI, which shows that delivery of BBR-loaded liposomes significantly improves their therapeutic availability [162].

### 4.4. Micelles

Micelles are surfactant-based complexes in which surface-active molecules are distributed in a bulk phase to produce micelles. Micelle formation commonly uses phospholipid mixtures of various phosphatidylcholines containing short and long hydrocarbon chains. Micelles can be spherical or discoidal in shape. Micelles can be used to solubilize medicines and, thus, improve their bioavailability, making them beneficial as pharmaceutical drug carriers [163]. Wang et al. developed anhydrous reverse micelles (ARMs), and the in vitro investigations revealed that the blood glucose levels (BGLs) of diabetic mice decreased by 57% after 4 h of oral administration of BBR-containing ARMs (100 mg/kg body weight), compared to BBR solution (2.5 mg/kg body weight), where BGLs of diabetic mice decreased by 22% after 4 h of intravenous administration; In addition, by encapsulating BBR in ARMs, the oral bioavailability of BBR was increased 2.4-fold [164]. Kwon et al. developed BBR-loaded mixed micelles, which enhanced BBR solubility and absorption permeability by 800% and 364%, and the efflux ratio reduced from 7.54 to 1.05, indicating that the P-glycoprotein-mediated efflux was inhibited, resulting in an increase in intestinal absorption of BBR [165].

### 4.5. Polymeric Nanoparticles

Polymeric nanoparticles are particulate dispersions or solid particles having a diameter ranging between 10 and 1000 nm. Particulate systems, such as nanoparticles, have been exploited to change and improve the pharmacodynamic and pharmacokinetic aspects of numerous medicinal compounds. These nanoparticles have been widely explored as nanocarriers in the medical and pharmaceutical areas, due to their regulated and sustained release characteristics, subcellular dimensions, and biocompatibility with tissues [166]. Vuddanda et al. developed polymeric nanoparticles of BBR chloride by a nanoprecipitation method, and the in vitro drug release of the F-6 (drug to polymer ratio: 1:3) formulation exhibited increased entrapment efficiency (by 82%) and increased bioavailability and promoted prolonged release [167]. Chang et al. developed polymeric nanoparticles (BBR/heparin/chitosan nanoparticle) using a simple ionic gelation technique, and the in vitro studies reveal that these polymeric nanoparticles exhibited controlled release in a simulated gastrointestinal dissolution medium. Moreover, in comparison to the BBR solution, these nanoparticles greatly enhanced the inhibition of *H. pylori* growth and significantly reduced the cytotoxic effects in *H. pylori*-infected cells [168]. Lu et al. developed an in situ forming gel based on simply combining carboxymethyl hexanoyl chitosan with low-molecular-weight hyaluronic acid. The gels formed were injectable, cytocompatible, and biodegradable and exhibited shape-persistent behavior and adhesive properties, and BBR was encapsulated within the gels. The gels exhibited a pH-responsive property that enabled them to release BBR in a sustained manner at pH 6.0 (simulating inflamed arthritic articular cartilage), and the degradation rates were accelerated at pH 7.4 (simulating healed normal tissue). The BBR-loaded gels effectively protected chondrocytes against sodium nitroprusside-induced apoptosis [169].

### 4.6. Dendrimer

Dendrimers are innovative polymeric nanoarchitectures with a hyper-branched three-dimensional structure and numerous functional groups on their surface, enhancing their efficiency and making them multipurpose and biocompatible. Their special characteristics, such as nanoscale uniform dimensions, the greater degree of branching, water-solubility, polyvalency, accessible interior spaces, and simple manufacturing methods, make them attractive biological and pharmacological delivery carriers. Dendrimers can be exploited to deliver a range of pharmaceutical substances. They can be utilized to improve pharmacological efficacy and reduce drug toxicity [170]. Gupta et al. covalently attached BBR to amine-terminated G4 poly (amidoamine) dendrimers and evaluated their characteristics in comparison with the BBR-loaded dendrimers. Both preparations had anticancer activities, with particle sizes ranging from 100 to 200 nanometers; however, the findings indicate that the conjugated formulation was more efficacious than the encapsulated one [171].

### 4.7. Carbon Nanotube

Carbon nanotubes (CNTs) are carbon allotropes composed of graphite and shaped into cylindrical tubes with nanometer-sized diameters and lengths of several millimeters [172]. CNTs are a potential platform for the delivery of therapeutic drugs because of their unique physicochemical characteristics. CNTs have been successfully used as a reliable delivery system for a variety of therapeutic active substances, including anti-infectives, cardiovascular medicines, genes, anti-neoplastic agents, and anti-inflammatory compounds, due to their huge potential [173]. Lohan et al. developed BBR-loaded multiwalled carbon nanotubes (MWCNTs) coated with phospholipid and polysorbate for the treatment of Alzheimer’s disease (AD). Factorial central composite design (FCCD) was used for systematic optimization. The selected optimized formulation possessed a particle size of 186 nm, 96% drug release in 16 h, and 68.6% drug adsorption, and the degree of carboxylation was found to be 36%. Basically, this research demonstrates the significant potential of polysorbate/phospholipid-coated MWCNTs of BRB in the overall management of AD [174].

**Table 2:** Summary of numerous nanoformulations, their dosage form, excipients, and outcomes in drug delivery of berberine (BBR).

**Technique**	**Dosage Form**	**Excipients**	**Outcomes**	**References**
**Polymeric Nanoparticles**				
Co-precipitation method	Nano-hydroxyapatite/chitosan (n-HA/CS) bone cement	Hydroxyapatite, chitosan, citric acid, calcium hydroxide, orthophosphoric acid, acetic acid, potassium dihydrogen phosphate, zinc oxide, calcium chloride	The n-HA/CS particles with BBR were effective in treating bone deformities, and n-HA/CS particles with 1 wt. % BBR were found to be an efficacious antibiotic drug delivery method.	[175]
Freeze-drying process	Fucose-chitosan/heparin nanoparticle	Sodium cyanoborohydride, fucose, chitosan, heparin, trehalose	These nanoparticles produced a prolonged BBR release at the site of infection as a result of their pH sensitivity, leading to an increased level of BBR in the mucus/epithelium layer and inhibiting *H. pylori* growth.	[176]
Ionic cross-linking method	Drug-loaded chitosan nanoparticles	Chitosan, sodium tripolyphosphate	BBR-loaded chitosan nanoparticles had a prolonged retention duration in synovial fluid and exhibited a stronger anti-apoptotic effect than free BBR in the treatment of osteoarthritis.	[177]
Solvent evaporation and freeze-drying	O-hexadecyl-dextran-entrapped BBR chloride nanoparticles (BC-HDD NPs)	Fetal bovine serum, rhodamine 123, dextran, sodium hydroxide,	BC-HDD NPs are as efficacious as BBR at reducing oxidative stress, apoptotic cell death, and mitochondrial depolarization when used at ~20-fold lower dose.	[178]
Emulsification method	PLGA nanoparticles	Polylactide glycolic acid (PLGA), didodecyl dimethyl ammonium bromide, polyvinyl alcohol (PVA)	The highest encapsulation efficiency (58%) of the nanoparticles was found at pH 8, using a water-immiscible solvent, dichloromethane	[179]
Solvent evaporation technique	Polymer–lipid hybrid nanoparticles	Soybean phosphatidylcholine, 4′,6-diamidino-2-phenylindole, polyethylene glycol	PEG–lipid–PLGA NPs/BBR–SPC’s oral bioavailability was dramatically increased by 343% after oral administration to rats in comparison to the suspension of BBR.	[180]
Emulsion solvent evaporation method	Polymeric nanoparticles	Sodium alginate, Tween 80	BBR-loaded polymeric nanoparticles had better antibacterial effectiveness than unloaded polymeric nanoparticles and BBR aqueous solution and were more effective against *Bacillus cereus* 240.	[181]
Ionic gelation method	Chitosan nanoparticles	Sodium tripolyphosphate, chitosan, glacial acetic acid	The combination use of chitosan nanoparticles and BBR provides synergistic action which allows for the efficient use of lower doses and increases their inhibitory effects against strains of *Bacillus subtilis* and *Staphylococcus aureus*	[182]
Dual emulsion method	PLGA polymeric nanoparticles	PLGA polymer, dichloromethane, polyvinyl alcohol	BBR-containing PLGA polymer nanoparticles had An encapsulation efficiency of 85.2% and will improve their efficiency on MCF-7 cancer cells.	[183]
Acid hydrolysis	Rod-shaped keratin nanoparticles (KNPs)	Hair, monopotassium phosphate, sodium chloride, disodium phosphate, potassium chloride	A large amount of BBR is released from KNPs at pH 1.2, showing that the photo-thermal action favors controlled release, and the NPs/BBR system exhibited anticancer activity against colon cancer cells	[184]
**Nanosuspension**				
High-pressure homogenization	Nanosuspension	Sodium lauryl sulfate, ceric ammonium nitrate, polyvinylpyrrolidone, azobisisobutyronitrile, calcium alginate, N,N-dimethylformamide	The administration of BBR nanosuspension-encapsulated HGFs greatly enhanced the healing process of *S. aureus*-infected wounds by its antibacterial action, stimulation of capillary development, and granulation, wound healing, hemostasis, and moisture regulation.	[185]
**Nanogel**				
Swelling/deswelling technique	Nanogel	Poly (diallyldimethylammonium chloride), Fluorescein diacetate, poly (allylamine hydrochloride), Carbopol Aqua SF1	Significant enhancement in antimicrobial activity at smaller incubation durations in comparison to non-coated nanogel particles loaded with BBR	[186]
**Solid lipid nanoparticle**				
High-pressure homogenization	Solid lipid nanoparticle	Propidium iodide, glyceryl monostearate, ethylene diamine tetraacetic acid, coumarin 6, paraformaldehyde, penicillin-streptomycin,	BBR-HCl-loaded SLNs had a significant influence on MCF-7 breast cancer cells compared to free BH in terms of lowering the growth rate and inducing arrest of cell cycle and apoptosis.	[152]
**Nanostructured lipid carriers**				
Hot melting followed by high-pressure homogenization	Nanostructured lipid carriers (NLCs)	Compritol 888, cremophor EL, d-α-tocopheryl polyethylene glycol 1000 succinate, oleic acid	BBR-NLCs effectively suppressed H22 cell growth, and in vivo testing revealed superior antitumor activity, with inhibition rates of 68.3%.	[156]
Hot homogenization and ultrasonication strategy	BBR-loaded NLCs overlaid with chitosan (BER-CTS-NLCs)	Poloxamer 407, glycerol monostearate, oleic acid	In comparison to BBR solution, BER-CTS-NLCs showed higher drug levels in the brain, showing that CTS-NLCs might be employed to target the brain via the intranasal route.	[187]
High-pressure homogenization	Drug-loaded NLCs	Compritol 888 ATO, olive oil, TPGS	BBR-NLCs, upon oral administration, greatly reduced colitis symptoms by inhibiting NF-κB nuclear translocation and lowering pro-inflammatory cytokine expression	[188]
Hot-melt dispersion/homogenization procedure	Selenium-coated NLCs	Sodium selenite, Precirol^®^ ATO 5, oleic acid	In comparison to regular NLCs and BBR solution, the BBR-loaded selenium-coated NLCs had a much better hypoglycemic action and also have a 6.63 times higher oral bioavailability than BBR solution.	[189]
**Reverse micelle**				
Lyophilization of water-in-oil emulsions	Anhydrous reverse micelle	Soybean phosphatidylcholine, medium-chain triglyceride	When compared to the BBR solution, the BER-loaded ARMs lowered diabetic mice’s blood glucose levels (BGLs) by 57% and increased oral bioavailability by a factor of 2.4.	[164]
**Liposomes**				
Ionophore A23187-mediated ZnSO4 gradient method	Liposomes	Hydrogenated soybean phospholipids, egg yolk lecithin, cholesterol, ammonium sulfate, soybean phospholipids,	The optimized liposomes of BBR hydrochloride have an encapsulation efficiency of 94.3 ± 2.1%.	[190]
Thin film hydration followed by sonication	Nano-liposome	Lecithin, chitosan, dihexadecyl phosphate	In the simulated gastrointestinal condition, chitosan-coated nano-liposomes showed superior stability and slower drug release than uncoated ones	[191]
Thin film hydration method	Liposome	Soyphsophatidylcholine, cholesterol	The vesicle diameter and entrapment efficiency results were reported to be extremely close to predicted values, and the observed particles are spherical with a zeta potential and an average diameter of −1.93 mV and 0.823 nm, respectively.	[192]

## 5. Conclusions

BBR has been one of the most widely explored isoquinoline alkaloids in recent years and is also employed in traditional Chinese medicine. It possesses a broad spectrum of pharmacological benefits, including anti-inflammatory, antihypertensive, antiarrhythmic, anti-Alzheimer, and anti-fibrotic effects, by interacting with a wide range of possible targets associated with the pathogenesis of the diseases. Despite its wide range of biological effects, its solubility, absorption, and bioavailability are limited. Nanotechnology, according to this review, is very beneficial in overcoming these obstacles. Different nanocarriers were found to have enhanced pharmacological properties of BBR. However, additional research is required in this area before nanoparticles may be used in clinics. 

## 6. Future Perspectives

The modern study of BBR’s pharmacological actions is flourishing, with countless scientific findings in the press and being presented at international conferences. The extraction or neo-synthesis of BBR analogs with better bioavailability is a possible future prospect. Furthermore, several researchers have assessed the clinical safety of BBR using acute, subacute, and sub-chronic toxicities. BBR has been shown in multiple clinical investigations to have low toxicity and no major adverse effects at standard doses. However, some individuals experience only minor gastrointestinal issues. Neurotoxicity, jaundice, and phototoxicity have also been reported in several studies. However, the severity of these side effects varied according to the dose, method, and length of therapy. BBR can potentially influence the effects of drug metabolic enzymes, which could lead to a drug–drug interaction. Nanotechnology can enhance the effectiveness and bioavailability of BBR due to reduced particle size, extremely large specific surface area, many active centers, greater surface reactivity, and great adsorption ability. It facilitates the targeted delivery of nano-drugs into hepatic cells, preventing the disruption of physiological activities in other organs.

## Figures and Tables

**Table 1:** The summary of the physicochemical properties and natural sources of berberine.

**Characteristic**	**Description**
Source	Family: Berberidaceae and genus: *Berberis* (~450–500 species) such as *Berberis vulgaris*, *Berberis aristata*, *Berberis lycium*, *Berberis buxifolia*, *Berberis chitria*, *Berberis darwinii.*
Form	Powder of chloride or sulfate salt
Color	Bright yellow
Taste	Bitter
Nature	Neutral
Solubility	Water-insoluble
Melting point	145 °C
Oral bioavailability	Low
Organ distribution	Highest concentration in the liver, followed by kidneys, muscle, lungs, brain, heart, and pancreas, and the lowest concentration in adipose tissue, which remains generally stable for 48 h
Metabolism	In liver, where it undergoes phase I demethylation before combining with sulfuric acid or glucuronic acid to produce phase II metabolites
Excretion	Feces (84%), urine (78%) and bile

## Data Availability

Not applicable.

## Abbreviations

α-SMA	α-smooth muscle actin
AChE	Acetylcholinesterase enzyme
AD	Alzheimer’s disease
AMPK	Adenosine 5′ monophosphate-activated protein kinase
ARMs	Anhydrous reverse micelle
BBR	Berberine
BGLs	Blood glucose levels
CHF	Congestive heart failure
CNTs	Carbon nanotubes
COX-2	Cyclooxygenase-2
FCCD	Factorial central composite design
HSC	Hepatic stellate cells
InsR	Insulin receptor
LDLR	Low-density lipoprotein-cholesterol receptor
MAO	Monoamine oxidase
MAPK/ERK	Mitogen-activated protein kinase/extracellular signal-regulated kinase
MFB	Myofibroblast-like cells
MMP-2	Matrix metalloproteinase
MWCNTs	Multiwalled carbon nanotubes
NAFLD	Non-alcoholic fatty liver disease
NLCs	Nanostructured lipid carriers
oxLDL	Oxidized low-density lipoprotein
PCOS	Polycystic ovary syndrome
PDGF-A	Platelet-related growth factor A
PGs	Prostaglandins
SLNs	Solid lipid nanoparticles
T2DM	Type 2 diabetes mellitus
TGF-β1	Transforming growth factor
TNF-α	Tumor necrosis factor-alpha
VSMCs	Vascular smooth muscle cells
